# Outcomes following staged bilateral total hip replacement: does first-side surgery predict the second?

**DOI:** 10.1308/rcsann.2022.0162

**Published:** 2023-08-17

**Authors:** U Jayaraju, J Boktor, V Joseph, S Yoganathan, M Elsheikh, PM Lewis

**Affiliations:** Cwn Taf Morgannwg University Health Board, UK

**Keywords:** THR, Oxford hip score, Bilateral, Outcome

## Abstract

**Introduction:**

Patient-reported outcome measures (PROMs) for bilateral staged total hip replacements (THRs) were reviewed to determine whether first-side surgery can predict second-side outcomes.

**Methods:**

A retrospective review was undertaken of a consecutive cohort of staged bilateral THRs using the same approach, implant and technique, from August 2009 to February 2020. Minimal important change (MIC) in PROMs was set at ≥5.

**Results:**

A total of 296 consecutive staged bilateral THRs were performed in 148 patients. Mean time interval between sides was 25 months (range 2–102). Mean age was 63.2 years for the first side and 65.3 years for the second; 62.8% of patients were female. Mean body mass index was 31.08 for the first side, increasing to 31.57 for the second side (*p* = 0.248). One-year follow-up PROMs were available for 96.6% and 92.5% of the first and second side, respectively. Mean PROMs improvement at 1 year was 26.4 for the first side and 25.1 for the second side (*p* = 0.207). Some 97.9% of patients achieved MIC for the first side and 96.3% for the second side (*p* = 0.092). Eight patients failed to reach an MIC on one side, all were female (*p* < 0.001); however, MIC was achieved for the contralateral side. Seven of eight patients (87.5%) achieved MIC by 2 years.

**Conclusions:**

This study identified no significant difference between first- and second-side PROMs improvements following staged bilateral THRs at 1-year follow-up. Failure to reach MIC on one side does not preclude success on the other. Female patients were more prone to not reach MIC at 1 year, but improvement was still subsequently achieved in the majority of cases. The informed consent process is able to reflect this expectation.

## Introduction

Elective orthopaedic surgery currently has the largest and longest waiting list in the National Health Service (NHS) (February 2022), with total hip replacements (THRs) among the most in-demand of all elective surgeries performed.^1,2^ In 2019, a total of 115,514 THRs were undertaken, as recorded on the National Joint Registry (NJR).^3^ Owing to increasing life expectancy, with patients presenting with multiple degenerative joints, there is growing demand for THRs, including second-side joint replacement surgery.^4^ Bilateral hip disease represents 42% of patients with osteoarthritis.^4^ Moreover, 25% of patients with hip osteoarthritis who are treated surgically will require bilateral staged or simultaneous THRs.^5^ Current practice in the UK shows that simultaneous bilateral THRs account for less than 0.5% of the relevant NJR data, with majority being staged bilateral THRs.^3^

To aid in surgical decision making and improve surgical outcomes, patient-reported outcome measures (PROMs) are utilised. PROMs 2021 report data, from NHS England, show that 97% of THRs improved based on their Oxford hip scores (OHS), with the average health gain on the OHS being 22.6.^6^ However, in comparison with this, only 95% of patients thought they felt ‘better’ and 93% considered their operative result to be excellent.^6^ These nationally reported improvements in OHS PROM scores do not take into consideration minimal important change (MIC). This concept relates to a patient reaching a minimum increase in their PROMs score, with improvement beyond error and to a meaningful level, that would be regarded as clinically significant rather than statistically significant.^7^

The aim of this study is to compare the outcomes of first- and second-side surgery in patients undergoing staged bilateral THRs. We evaluate PROMs improvement, MIC, body mass index (BMI) and intraoperative/postoperative complications to determine whether first-side outcomes predict second-side outcomes. These results can then be utilised during the consent process to guide expectations, as per General Medical Council guidelines, for the large cohort of patients undergoing second-side surgery.^8^

## Methods

A retrospective review of a prospectively updated single-surgeon database was undertaken to evaluate a consecutive series of staged bilateral THRs performed between August 2009 and February 2020. Each patient’s OHS was recorded preoperatively, at 6 weeks and 1 year postoperatively. MIC was set at an OHS change ≥5, as per Beard *et al*.^7^

### Inclusion criteria

All staged bilateral THRs used the same approach (standard posterior hip approach), same technique, same lead surgeon and same implant system (Cementless Corail/Pinnacle system, DePuy Synthes). Minimal follow-up was 1 year.

### Exclusion criteria

Patients who had undergone unilateral THR, revision THR, cemented THR or were receiving special implants were excluded from the study.

### Data extraction

All data were collected prospectively, as per surgical documentations, including postoperative complications and long-term follow-up. Our database was analysed against operative and patients’ records on an online hospital system. Ethical approval was not required because this was a review of routinely collected prospective information. Approval for the study was, however, sought and granted through the research and development department of the health board.

### Research question

The research question was: Do patient-reported outcome measure scores (PROMs) from first-side surgery predict the results of the second side in staged bilateral THRs?

### Outcomes

The primary outcome was the mean improvement in OHS between first- and second-side surgery in bilateral staged THRs. Additional analysis was undertaken to evaluate the MIC for each side and patient demographics, which may influence overall outcomes.

Secondary outcomes were to review differences in implant sizes within the same patient. Further comprehensive analysis was undertaken of patients not achieving MIC on one side compared with the other side, as well as compared with the whole cohort, and any correlation between complications and PROMs for first- and second-side surgery. We also examined mean improvement and MIC difference in PROMs between patients’ second side vs the concurrent cohort of unilateral THRs performed by the same surgeon, utilising the same technique and same implants over the same period.

### Statistical analysis

The anonymised data set was analysed utilising SPSS Statistics software version 27 (IBM, New York, US). Continuous variables were analysed using paired *t*-test or independent sample *t*-test where indicated and chi-squared test was used for categorical variables.

## Results

**Figure 1:**
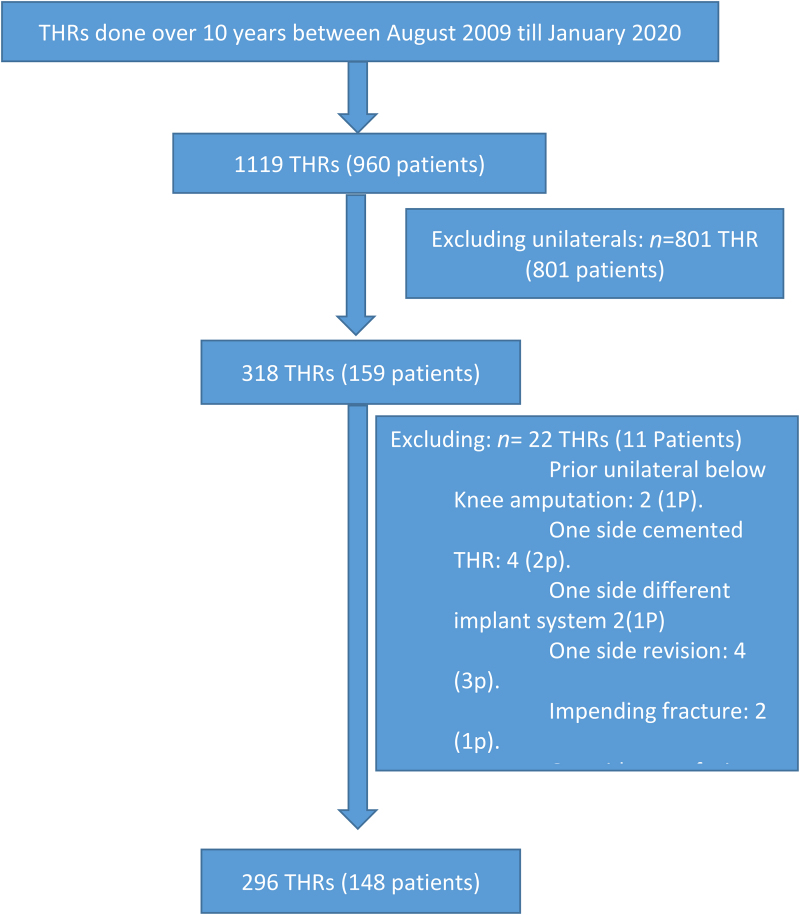
All total hip replacements done by same surgeon over study period and selection of the study group and reasons for exclusions

A total of 148 consecutive non-simultaneous, staged bilateral THR patients were included (296 joints), from a cohort of 159 bilateral procedures (318 joints) and 801 unilateral elective primary procedures performed over the concurrent study period. Reasons for the exclusion of 11 patients with bilateral THRs are in Figure 1.

Patient demographics are shown in Table 1. The mean age for patients undergoing first- and second-side surgery was 63.2 years (range 25–86) and 65.3 years (range 27–87), respectively (Table 3). The majority of these patients were female (*n* = 93/148, 62.8%) and aged 61 years or above (*n* = 98/148, 66.2%). In all, 50% of patients underwent left-side surgery first. BMI was >30 at the time of first surgery in 54.7% of cases (*n* = 81/148) (Table 3). The operative indication was predominantly osteoarthritis (*n* = 143/148 [96.6%]), with remaining causes being: adult dysplasia in three cases (2.0%) and Perthes' disease in two cases (1.4%). The mean interval before undertaking second-side surgery was 24 months (range 2–102) (Table 2). Mean BMI increased between the first (mean = 31.08kg/m^2^, range 20–52) and second side (mean = 31.57kg/m^2^, range 20–54) but was statistically insignificant (*p* = 0.248) (Table 3).

**Table 1:** Patient demographics

	No. of patients (%) (*n* = 148)
Sex	
Male	55 (37.2)
Female	93 (62.8)
Age at time of first side	
<40	4 (2.7)
40–60	46 (31.1)
61–80	92 (62.2)
>80	6 (4.1)
First-side laterality	
Right	74 (50.0)
Left	74 (50/0)
BMI	
<25	23 (15.5)
25–30	40 (27.0)
31–40	68 (46.0)
>40	13 (8.8)
N/A	4 (2.7)
Reasons for surgery	
OA	143 (96.6)
Perthes	2 (1.4)
Adult dysplasia	3 (2.0)
ASA grade	
1	30 (20.3)
2	42 (28.4)
3	64 (43.2)
4	12 (8.1)

ASA = American Society of Anaesthesiologists; BMI = body mass index; N/A = not available; OA = osteoarthritis

**Table 2:** Time interval between staged total hip replacements

Interval between first and second side (months)	Number (%) (*n* = 148)
< 6	5 (3.4)
6–12	33 (22.3)
> 12	110 (74.3)

**Table 3:** Mean BMI and age changes for first- and second-side total hip replacements

	At time of first side	At time of second side
BMI	Mean = 31.08, range = 20–52	Mean = 31.57, range = 20–54; *p* = 0.248
Age (years)	Mean = 63.2, range = 25–86	Mean = 65.3, range = 26–87

BMI = body mass index

**Figure 2:**
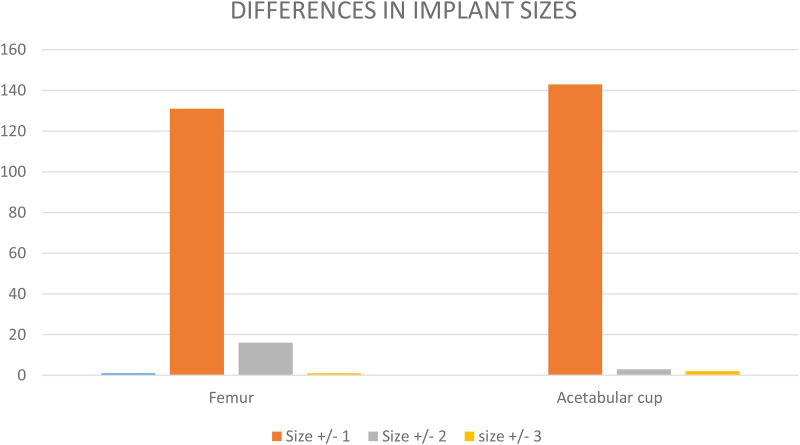
Implant size differences in the whole cohort

Regarding implant size differences, for the femoral stem, stem size difference was 0 ± 1 in 131/148 patients (88.5%), 0 ± 2 in 16/148 (10.8%) and 0 ± 3 in the remaining 1/148 (0.7%). For the acetabular component, the size difference was 0 ± 1 in 143/148 (96.6%), 0 ± 2 in 3/148 (2.3%) and 0 ± 3 in the remaining 2/148 (1.4%). (Figure 2).

### Follow-up

Complete PROMs data were available for 96.6% (*n* = 143/148) of patients following first-side surgery and 92.6% (*n* = 137/148) following second-side surgery. At 1 year, the mean improvement in OHS was 26.4, with 97.9% (*n* = 140/143) achieving an MIC following first-side surgery. Following second-side surgery, mean OHS improvement was 25.1 (*p =* 0.207) with 96.4% (*n* = 132/137) achieving MIC (*p =* 0.092) (Table 4).

**Table 4:** Mean follow-up PROMs at first and second side at 1 year

THR	No. of responses (%) (*n* = 148)	Mean change in PROMs at 1 year (preoperative to 1 year)	MIC achieved [mean change in PROMs score]	MIC not achieved
First side	143 (96.6)	26.4	97.9% (*n* = 140/143)	2.1% (*n* = 3/143)
Second side	137 (92.6)	25.1 *p =* 0.207	96.4% (*n* = 132/137) [27.2] *p =* 0.092	3.7% (*n* = 5/137) *p =* 0.246

MIC = minimal important change; PROM = patient-reported outcome measure; THR = total hip replacement

Of the three patients not initially achieving MIC at 1 year following first-side surgery, two did achieve an MIC before proceeding for the second-side procedure at 2 years follow-up and they had the operation at a 26-month interval from the first. The third patient, although not achieving an MIC following their first side, chose to proceed with the other side because it was the main cause of pain and limited daily activity. None of these three patients suffered any intraoperative or postoperative complication. Five patients did not achieve MIC at 1 year following their second-side surgery. However, they did achieve it following their first-side surgery. Again, none of these patients suffered any intraoperative complications, but one patient subsequently suffered a postoperative complication of THR dislocation (Table 5). The mean time interval between the first- and second-side surgery for these five patients was 17 months (range 9 to 54), which is less than the whole cohort mean of 24 months (range–102) (*p* = 0.721).

**Table 5:** Complications for both sides

	Complications		MIC not achieved	Death early postoperative
	Intraoperative (*n* = 148)	Postoperative in 1 year (*n* = 148)		
First side	None	*n* = 3 (2.0%) Haematoma (*n* = 2, 1.4%) Multiple leg ulcers (*n* = 1, 0.7%)	None	N/A
Second side	None	*n* = 3 (2.0%) Transient femoral nerve palsy (*n* = 1, 0.7%) Multiple dislocations (*n* = 1, 0.7%) Multiple leg ulcers (*n* = 1, 0.7%)	Multiple dislocations (*n* = 1, 0.7%)	GIT aspiration (*n* = 1, 0.7%)

GIT = gastrointestinal tract; MIC = minimal important change; N/A = not available

There were a total of six complications for the entire cohort of 296 THRs (2.0%), with only one of these six (16.7%) not achieving an MIC at 1 year although they did achieve it at 2-year follow-up. One patient died within 3 months of their postoperative period following second-side surgery, from aspiration pneumonia (Table 5).

**Figure 3:**
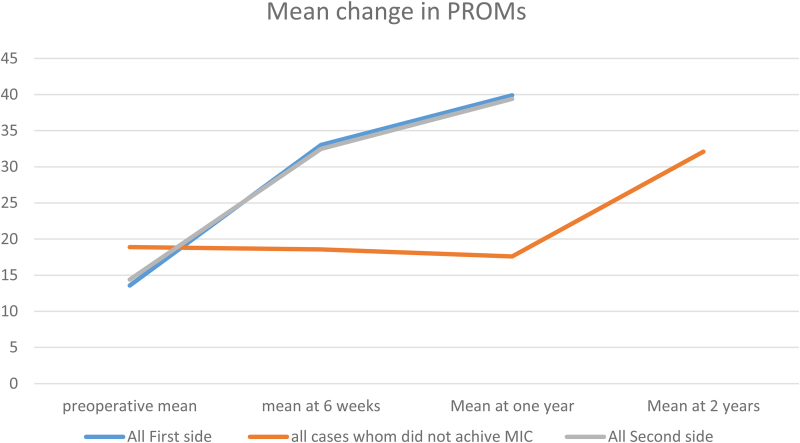
Change in mean patient-reported outcome measures at different time points for different groups

Further analysis of the eight patients not achieving MIC on one side by 1-year follow-up revealed that all were female; in comparison, females represent 62.8% (93/148) of the entire cohort (*p* < 0.001). Recorded BMI for the side that achieved MIC was 32.3, and this was greater on the side that did not, with a BMI of 32.3, although this did not reach significance (*p* = 0.189) (Table 6). On detailed review of MIC at different time points, three of the eight patients (37.5%) achieved MIC at < 6 months. On further follow-up, seven of the eight patients (87.5%) achieved an MIC in their PROMs by 2 years of follow-up (Figure 3). Regarding differences in implant sizes for those patients who did not achieve MIC, six of the eight patients had (±1) femoral stem size difference, and all eight had (±1) acetabular cup size difference, between sides (Table 6).

**Table 6:** Differences between sides in patient not achieving MIC

	Side achieved MIC (*n* = 8)	Side not achieved MIC (*n* = 8)
Age	64.35 (SD 11.7)	62.6 (SD 8.70) (*p* = 0.339)
BMI	31.3 (SD 6.34)	32.3 (SD 8.06) (*p* = 0.189)
Laterality		
Right	6 (75.0%)	
Left	2 (25.0%) (*p* = 0.346)	
Cup size		
Equal	5 (62.5%)	
Smaller (±2)	3 (37.5%)	
Cup screws	3 (37.5%)	
Femoral stem size		
Equal	4 (50.0%)	
Larger	4 (50.0%)	

BMI = body mass index; MIC = minimal important change

**Figure 4:**
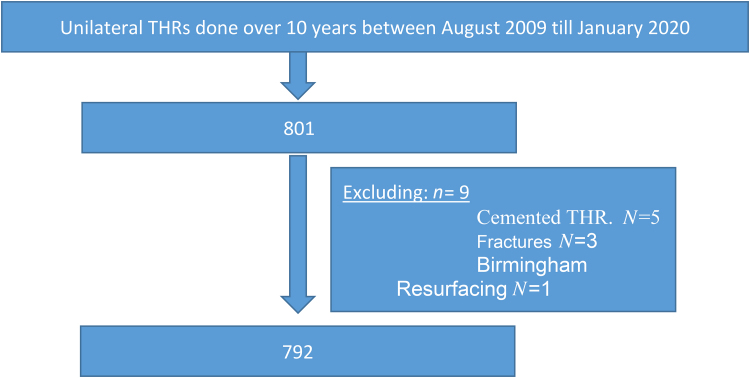
Unilateral total hip replacements included in secondary outcome analysis

Reviewing secondary outcomes, we compared second-side PROMs against all unilateral THRs performed concurrently by the same team with the same implant and with exclusions as per methodology protocol (Figure 4). The mean change in PROMs at 6 weeks and 1 year for unilateral THRs were 19.3 and 23.6, respectively, whereas for second-side bilateral staged THRs, OHS was 18 at 6 weeks (*p* = 0.296) and 25.1 at 1 year (*p* = 0.866). In total, 95.1% achieved MIC in the unilateral THR group compared with 95.6% reaching MIC for the second-side surgery in the bilateral staged group (*p* = 0.77) (Table 7).

**Table 7:** Mean follow-up PROMs at second side in staged bilateral and unilateral at 6 weeks and 1 and 2 years

THR	Response at 6 weeks	Response at 1 year	Mean change in PROMs (preoperative to 6 weeks)	Mean change in PROMs (preoperative to 1 year)	MIC achieved [mean change in 1 year]	MIC not achieved [mean change in 1 year]
Unilateral	90.9% (*n* = 720/792)	74.2% (*n* = 588/792)	19.3	23.66	79.8% (*n* = 469/588) [25.13]	4.9% (*n* = 29/588) [0.93]
Second-side staged bilateral	85.1% (*n* = 126/148)	92.6% (*n* = 137/148)	18 (*p =* 0.296)	25.1 (*p =* 0.866)	96.3% (*n* = 131/136) [26.3] (*p* = 0.77)	3.6% (*n* = 5/136) [0.2] (*p* = 0.377)

MIC = minimal important change; PROM = patient-reported outcome measure

## Discussion

Approximately one million THRs are performed globally each year for patients with advanced hip OA, with an estimated one in five patients going on to require contralateral side hip surgery.^4,9^ Following the Montgomery case of 2015, the General Medical Council introduced new consent principles for doctors and patients during the informed decision process.^8^ This study addresses patient expectations when undergoing second-side or staged bilateral THRs. We have been able to demonstrate that there is no significant difference between first- and second-side THR surgery during bilateral staged procedures by reviewing detailed patient demographics and PROMs. In addition, in the few patients failing to show a clinically meaningful improvement in their PROMs following first-side surgery, the contralateral side can still reach this threshold. This is of particular importance for patients making a decision on proceeding with second-side surgery.

This retrospective review of a prospectively updated surgical database analysed a 10-year period of practice from a single surgeon using a standard technique and implants for all patients undergoing primary staged bilateral THRs. Reinforcing the recognised successful outcomes following hip replacement surgery,^10^ we identified 97% of patients achieving an MIC at 1-year follow-up whether this was first- or second-side surgery. The mean improvement in OHS was 26.4 following first-side surgery and 25.1 for the second side. No significant difference was identified between first- and second-side PROMs improvements (*p =* 0.207), with both sides comparing favourably with the reported national PROMs mean improvement of 22.6.^6^

A retrospective review from Belfast in 2021, again using a standardised technique, implant and OHS PROMs, revealed similar findings to our results: 119/122 (97.5%) patients had an improvement greater than the MIC for first-side surgery, whereas the second side showed an improvement in 121/122 (99.2%) patients. A total of 1.6% patients did not achieve MIC for one side of their staged bilateral THRs.^9^ Using the same delta gain threshold of a five-point MIC, our study showed similar results with only 3.0% (*n* = 9/296) not reaching MIC on one side at 1 year, improving to 1.7% (*n* = 5/296) at 2-year follow-up.

In our review, the total number of complications was small and not predictive of similar issues for the opposite side. Of the eight patients not reaching MIC on one side, all achieved an MIC for their second side. Only one of the eight cases (12.5%) suffered a significant complication with multiple dislocations within 6 months requiring revision for bearing exchange, and subsequently still reached MIC at 2 years. We found no obvious attributable or confounding factors to cause this complication. BMI and changes in BMI between surgeries, age and differences in implant size did not appear to influence outcomes. Sex of the patient, however, in those that failed to achieve MIC did appear significant, with all eight affected patients being female (8.6%; *n* = 8/93), whereas all male patients achieved MIC by 1 year (100%, *n* = 55/55) (*p* < 0.001).

Within the literature there is evidence correlating gender and OHS outcomes at 1 year. Warnock *et al*, in 2019, investigated whether there was a correlation between OHS changes and gender-specific findings in a cohort of 123 patients (75 females, 48 males). It was noted that females received a smaller femoral implant leading to a bias toward a conservative (higher) neck cuts compared with males and potential femoral lengthening. However, the study suggested that males have a greater acetabular floor depth compared with females and thus reaming to the true floor results in greater loss of acetabular offset. There does not, however, appear to be an obvious explanatory gender difference in our cohort.^11^

Further relevant literature correlates stress and anxiety influencing OHS, where females are more prone to preoperative stress that will influence early postoperative results, yet the study did not comment on longer-term follow-up outcomes.^12^ Rolfson *et al* studied data from the Swedish Hip Arthroplasty Register in 2009, which included 6,158 patients with primary OA of the hip, analysing gender and anxiety effect. They concluded that females had worse outcome scores than males. More interestingly, only 24% of the patients in the persistent anxiety/depression group improved in the mobility dimension, compared with 59% in the remaining group.^13^ In 2020, Graham *et al* prospectively collected data for 1,384 patients undergoing THRs in Singapore and reviewed their OHS. They stratified patients into two categories: with psychological stress and without psychological stress. Graham *et al* found that distressed patients had a poorer physical component at 6-month follow-up, yet at 2 years there was no statistical difference in OHS. Our study showed similar outcomes, with seven of the eight patients who did not reach MIC at 1 year reaching MIC at 2 years.^14^

Within the secondary analysis, no difference was identified between second-side staged bilateral THRs and the unilateral THR cohort either in mean PROMs changes at 1 year or in the percentage not achieving a 1-year MIC. We believe this is the first study to carry out such a comparison with a large cohort over this period.

Regarding implant size differences, it is noted that using the (0 ± 1) formula covered 96.6% of acetabular cup (143/148) and 88.51% of femoral stems (131/148). This represents a potential for surgical tray rationalisation (STR). The STR principle consists of a systematic reduction in the number of surgical instruments used to perform specific procedures without compromising patient safety while reducing losses in the sterilisation and assembly of trays.^15^ Utilised alongside traditional templating methods, there is an opportunity to optimise surgical instrument trays, improving theatre efficiency, reducing costs, aiding implant procurement plus the practical benefit in reducing the weight of trays.^16^ Although implant size difference was not a prime indication for the study or a secondary outcome, this is a finding that can be used for future research.

### Limitations of the study

There are some limitations to this study. This is a retrospective data review, albeit of prospectively collected data, and data collection was high (number of patients and return of data). It was also a single-centre, single-surgeon, single-component system and technique, with a relatively limited number of cases owing to the inclusion criteria of only staged bilateral cases. There is no clear consensus on the defined timing of a ‘staged bilateral procedure’ compared with two independent interventions. A recently published study utilised ±2 SD from the mean of the original whole cohort for inclusion of patients as having undergone a staged bilateral procedure.^9^ In our study, the intervals between procedures were wide and with a standard deviation of 20 months and hence using ±2 standard deviation would not influence the results. Finally, we analysed only OHS PROMs, and make no comment on pain visual analogue score or patients’ opinions on surgery.

## Conclusions

This large single-surgeon cohort identified no significant difference between first- and second-side OHS improvements following staged bilateral THRs at 1-year follow-up. MIC was achieved in 97.9% of first-side surgeries and 96.3% of second-side surgeries. Failure to reach MIC on one side does not predict failure of the other. Females appear more prone to not reaching MIC for one side at 1 year, although even within this small subsection, the majority can still achieve a successful outcome and MIC in PROMS by 2 years. The informed consent process is therefore able to reflect this expectation.
